# Surveillance Strategy after Curative Resection for Oesophageal Squamous Cell Cancer Using the Hazard Function

**DOI:** 10.1186/s12885-022-10345-5

**Published:** 2022-12-01

**Authors:** Kyohei Kanematsu, Yozo Kudose, Daichi Utsunomiya, Kentaro Kubo, Yusuke Fujii, Daisuke Kurita, Koshiro Ishiyama, Junya Oguma, Hiroyuki Daiko

**Affiliations:** 1 Department of Oesophageal Surgery, National Cancer Center Hospital, 5-5-1 Tsukiji, Chuo-ku, Tokyo, 104-0045 Japan; 2 Course of Advanced Clinical Research of Cancer, Juntendo University Graduate School of Medicine, Tokyo, Japan

**Keywords:** Oesophageal squamous cell cancer, Hazard rates, Hazard function, Recurrence, surveillance

## Abstract

**Background:**

The optimal surveillance period and frequency after curative resection for oesophageal squamous cell carcinoma (OSCC) remain unclear, and current guidelines are mainly based on traditional Kaplan–Meier analyses of cumulative incidence rather than risk analysis. The aim of this study was to determine a suitable follow-up surveillance program following oesophagectomy for OSCC using the hazard function.

**Methods:**

A total of 1187 patients who underwent curative resection for OSCC between 2000 and 2014 were retrospectively analyzed. The changes in the estimated hazard rates (HRs) of recurrence over time were analyzed according to tumour-node-metastasis stage.

**Results:**

Four hundred seventy-eight (40.2%) patients experienced recurrence during the follow-up period (median, 116.5 months). The risk of recurrence peaked at 9.2 months after treatment (HR = 0.0219) and then decreased to half the peak value at 24 months post-surgery. The HRs for Stage I and II patients were low (< 0.007) post-treatment. The HR for Stage III patients peaked at 9.9 months (HR = 0.031) and the hazard curve declined to a plateau at 30 months. Furthermore, the HR peaked at 10.8 months (HR = 0.052) in Stage IV patients and then gradually declined from 50 months.

**Conclusions:**

According to tumour-node-metastasis stage, changes in the HRs of postoperative recurrence in OSCC varied significantly. Intensive surveillance should be undertaken for 3 years in Stage III patients and for 4 years in Stage IV patients, followed by annual screening. For Stage I OSCC patients, a reduction in the surveillance intensity could be taken into consideration.

**Supplementary Information:**

The online version contains supplementary material available at 10.1186/s12885-022-10345-5.

## Introduction

The multimodal treatment of oesophageal cancer (OC) has developed over the past few decades toward precise surgical technique and combination of perioperative chemoradiation therapy. However, even after curative resection, recurrence often occurs in patients. The early detection of treatable recurrence through appropriate postoperative surveillance can offer the chance of cure [1]. Several series have demonstrated that most recurrences occur in the first 2 years after the completion of treatment [2, 3], but there is scant evidence on the optimal frequency of surveillance after successful treatment of OC. Most of the major guidelines for OC do not mention the optimal postoperative surveillance after curative treatment for OC. Only the National Comprehensive Cancer Network (NCCN) guidelines propose a detailed postoperative surveillance program according to pathological stage. For patients with Stage II or III OC, CT scans are recommended every 6 months for up to 2 years. However, for patients with pT1b tumours, surveillance with annual CT scans up to 3 years is acceptable [4].

Many of the literatures adopted by the NCCN guidelines is mostly based on the evidence of cumulative incidence using the Kaplan-Meier method. The Kaplan-Meier method indicates ﻿the cumulative incidence at any given time point for all eligible patients and ﻿does not directly reflect the risk of an event at a specific time point. To consider optimal postoperative surveillance, evaluating the peak time of recurrence hazard and recurrence hazard changes over time is essential, as previously reported in other cancer types [5–7]. The hazard function conveys ﻿continuous estimation of the hazard rates (HRs) over time for the risk of an event among only those patients remaining at risk at a specific time point [8, 9].

To determine an evidence-based timetable for postoperative surveillance, we focused on the changes in the estimated HRs for recurrence over time using the hazard function. This knowledge would enable us to make decisions regarding the interval and intensity of surveillance. We analyzed the transition of recurrence hazard and the peak recurrence time of patients stratified by tumour-node-metastasis (TNM) stage. We hypothesized that OC of higher stage shows a higher hazard rate of recurrence and shorter peak time, thus requires more intensive follow-up. The ultimate purpose of this study was to ascertain a rational follow-up schedule after curative surgery for OC.

## Patients and methods

### Patients

We conducted a single-institution, retrospective cohort study in which we reviewed patients who had undergone esophagectomy for thoracic oesophageal squamous cell carcinoma (OSCC) at the National Cancer Center Hospital (NCCH), Japan, between January 2000 and December 2014. Patients who underwent R2 resection and patients with histological types other than squamous cell carcinoma were excluded. We extracted following parameters from a prospectively maintained database: age, sex, tumour location, histology, depth of tumour, presence of lymph node metastasis, pathological stage according to Union for International Cancer Control tumour-node-metastasis classification (eighth edition) [10], details of treatment, postoperative recurrence and vital status.

### Surgery

﻿Surgery was performed by the same experienced surgical team at our institute during the study period. All patients underwent transthoracic esophagectomy with two or three-field nodal dissection of the neck, mediastinum, and abdomen. Regional lymph node dissection comprised removal of the mediastinal lymph nodes including lymph nodes along the bilateral recurrent nerve and perigastric and celiac lymph nodes. Reconstruction of the alimentary tract was performed with a gastric tube, the colon or the jejunum with cervical or intrathoracic anastomosis. The surgical approach had remained mostly unchanged except for the application of thoracoscopy or laparoscopy.

### Neoadjuvant Therapy

﻿During the study period, drastic changes in the therapeutic strategy for advanced OC have occurred worldwide and neoadjuvant therapy had become the standard of care. In Japan, unlike western countries, where chemoradiation therapy is the standard treatment as a neoadjuvant therapy for OC, neoadjuvant chemotherapy is the mainstream approach for advanced OSCC to ensure early control of microscopic metastasis. Patients with cStage II or higher OSCC were treated with neoadjuvant chemotherapy 5-FU/cisplatin since 2007 based on the JCOG9907 trial as a standard treatment [11]. Patients with advanced OC who were deemed medically unfit for adjuvant or neoadjuvant chemotherapy received only surgery. In this study, 438 patients (cStage II:165; cStage III:207; cStageIV: 47) received neoadjuvant chemotherapy.

### Postoperative Follow-up Schedule

Postoperative surveillance of patients at the NCCH was performed according to the surveillance methods defined at our hospital. In practice, physical examinations and measurements of serum tumour markers and CT scans of the neck, chest, and abdomen with intravenous contrast were performed every 4 months during the first year, and then annually performed up to the tenth year after primary surgery. Upper gastrointestinal endoscopy was performed every year after surgery. Recurrence was diagnosed on the basis of the appearance of new lesions on CT or positron emission tomography images or histological findings through biopsy. Recurrence status was censored on the date of the last hospital visit.

### Statistical Analysis

Overall survival (OS) was defined as the time interval between the start of neoadjuvant chemotherapy or surgery and the date of death from any causes until 31 December 2020 or last follow-up. Recurrence-free survival (RFS) were determined from the start of the initial treatment to the date of detection of first recurrence, last follow-up or death from any cause until 31 December 2020. Survival was calculated according to the Kaplan–Meier method. In addition, we focused on the analysis of continuous estimation of the HR over time, which offered visualized chronological changes of risk for tumour recurrence. The HRs over time were estimated by the non-parametric method proposed by Muller and Wang using the kernel smoothing method [12]. All statistical analyses were performed using R software (version 4.0.2).

The study was approved by the NCCH Ethics Committee (registration no.2017–061).

## Results

### Patient and Clinicopathologic Characteristics

**Fig. 1:**
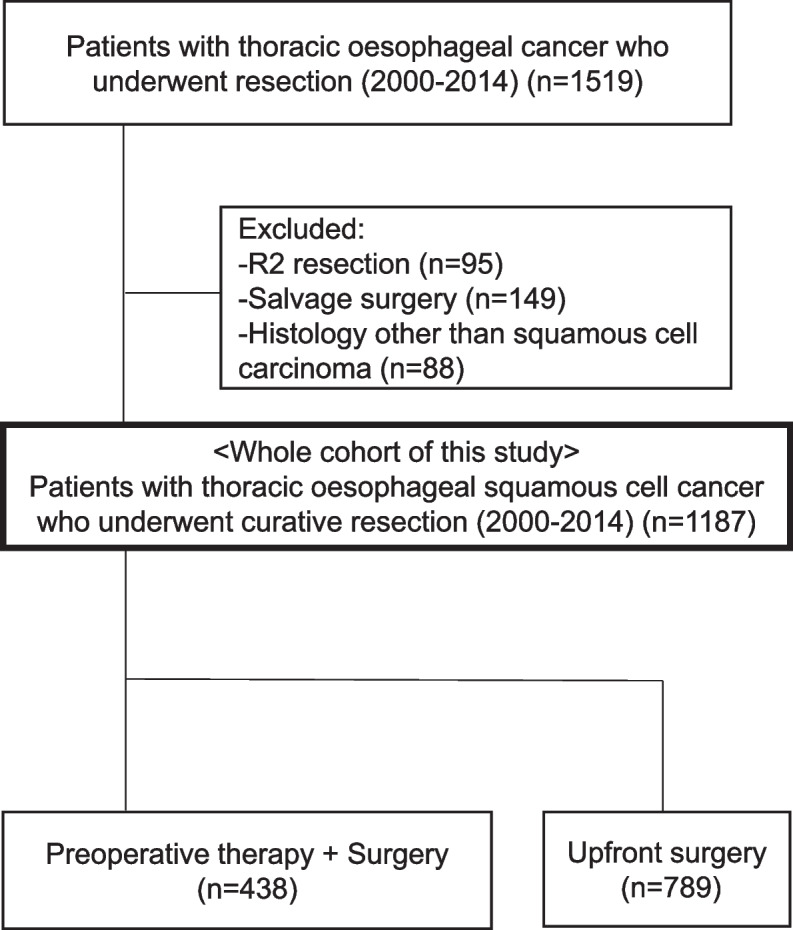
Patient selection and exclusion criteria

Of the 1519 patients who underwent esophagectomy for thoracic OC at the NCCH between 2000 and 2014, 1187 patients met the selection criteria. Figure 1 shows the flowchart of patient selection. We excluded R2 resection (*n* = 95), salvage surgery after definitive chemoradiotherapy (*n* = 149) and histological type other than squamous cell carcinoma (*n* = 88). The clinical and pathological characteristics of these patients are provided in Table 1. Follow-up was performed until December 2020. The median follow-up period for those alive and without recurrence at the study end was 116.5 months (range, 4.5–248 months). A total of 437 patients (36.8%) had received neoadjuvant therapy before surgery and 124 patients (10.4%) had received adjuvant therapy after surgery. Six hundred twenty-six patients (52.7%) underwent surgery alone. Only 6.7% of the patients (*n* = 79) had R1 resection. During the review period, 478 patients (40.2%) had developed a recurrence.

**Table 1:** Patient Characteristics (n = 1187)

Category	Number (%)
Gender	
Male	1054(88.8)
Female	133(11.2)
Age(yr)	
Median (range)	64(30-87)
Differentiation	
G1	259(21.8)
G2	454(38.2)
G3	311(26.2)
GX	163(23.7)
Adjuvant therapy	
Induction	438(36.8)
No	749(63.1)
Clinical stage	
I	154(13.0)
II	410(34.5)
III	455(38.3)
IVA	61(5.1)
IVB	107(9.0)
Pathological stage	
0 (pTisN0M0)	3(0.3)
I	267(22.5)
II	209(17.6)
IIIA	114(9.6)
IIIB	344(29.0)
IVA	123(10.4)
IVB	127(10.7)
Location	
Upper third	169(14.2)
Middle third	553(46.6)
Lower third/Cardia	465(39.2)
Margins	
R0	1108(93.3)
R1	79(6.7)
﻿Number of lymph node examined	
﻿ Median (range)	55(12-139)

### Recurrence-Free Survival and Hazard Rates of Recurrence for All Patients

**Fig. 2:**
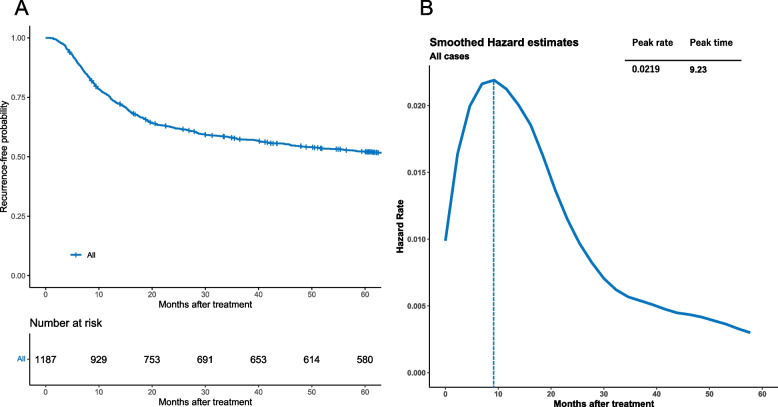
Kaplan-Meier plot (with 95% confidence intervals) of the time to recurrence in all patients (**A**) and smoothed hazard functions for recurrence in all patients (**B**)

Figure 2A shows the traditional Kaplan–Meier estimate of the RFS (with 95% confidence intervals) of 1187 patients enrolled in this study. ﻿The total number of events was 640 (RFS), and RFS consisted of 162 cases of death and 478 cases of disease recurrence. The survival plotting of HR over time revealed that the risk of recurrence increased steeply towards a peak (HR = 0.0219) 9.2 months after treatment, after which it decreased to less than half the peak value at 24 months (Fig. 2B).

### Overall Survival and Hazard Rates of Death from Any Cause Stratified by TNM Stage

**Fig. 3:**
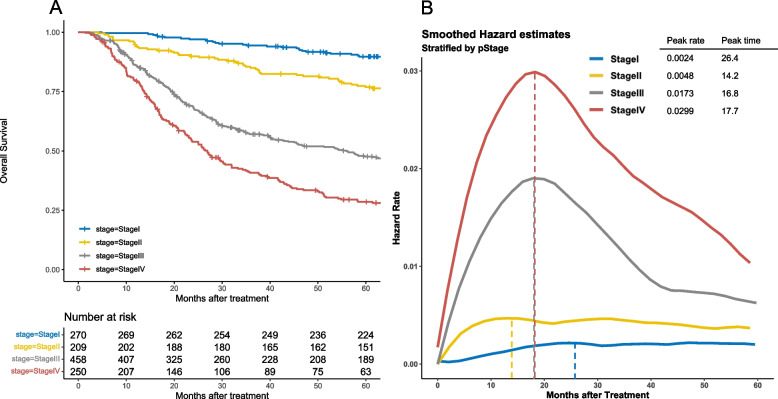
Kaplan-Meier plots of the time to recurrence stratified by TNM stage in all patients (**A**) and smoothed hazard functions for recurrence stratified by TNM stage in all patients (**B**)

All patients were divided into groups according to TNM stage [10]. Figure 3A shows OS curves stratified by TNM Stage. Hazard rates of death from any cause stratified by TNM stage are shown in Fig. 3B. Hazard rates for stage III and IV OSCC peaked at about the same time (16.8 and 17.7 months after treatment, respectively), although peak hazard rates differed significantly (0.00173 and 0.0299, respectively). Hazard rates for Stage I and II showed no distinct peak. At any time point, higher stage OSCC showed higher hazard rates than lower stage OSCC.

### Recurrence-Free Survival and Hazard Rates of Recurrence for All Patients Stratified by TNM Stage

**Fig. 4:**
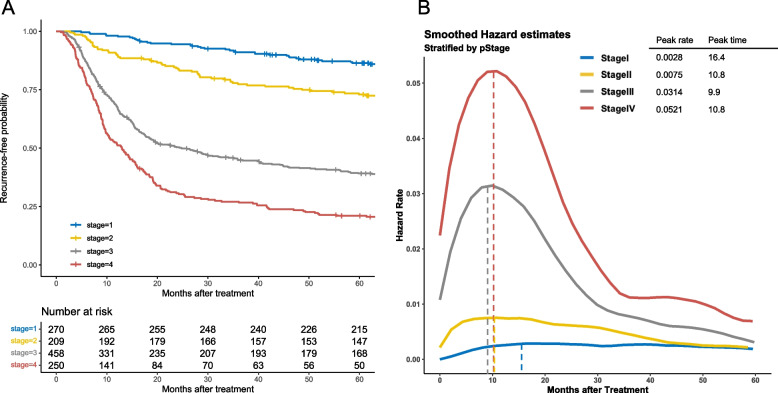
Smoothed hazard functions for recurrence stratified by the presence or absence of neoadjuvant therapy among patients with (**A**) stage I, (**B**) stage II, (**C**) stage III, and (**D**) stage IV oesophageal squamous cell carcinoma

Recurrence-free survival curves of all patients stratified by TNM stage are shown in Fig. 4A. ﻿Five-year RFS percentages were 86.4% (*n* = 217) for Stage I, 72.9% (*n* = 148) for Stage II, 39.1% (*n* = 169) for Stage III, and 20.6% (*n* = 49) for Stage IV. After stratification by TNM stage, HRs were plotted ﻿against time (Fig. 4B). This analysis offered visualized dynamics of recurrence according to TNM stage. HRs for Stage I OSCC was consistently low (less than 0.003), and the curve showed no apparent peak. In Stage II OSCC, the peak value of HRs was twice as high as Stage I OSCC and the curve showed no prominent peak. From 45 months, HRs become the same value as in Stage I. The HR of recurrence for patients with Stage III OSCC increased more steeply than that of patients with Stage I and II OSCC and peaked at 9.9 months (peak HR: 0.0314), followed by a decrease with a long slope to the right. The HRs for patients with Stage IV OSCC were consistently higher than HRs for patients with Stage III OSCC throughout the surveillance period. The peak time for HRs showed no distinct difference between the stages of OSCC. The figure of hazard curves for recurrence has different shapes from that for death from any cause in each stage.

### Hazard Rates of Recurrence Stratified by TNM Stage among Patients with Surgery Alone and Patients with Neoadjuvant Therapy Followed by Surgery

**Fig. 5:**
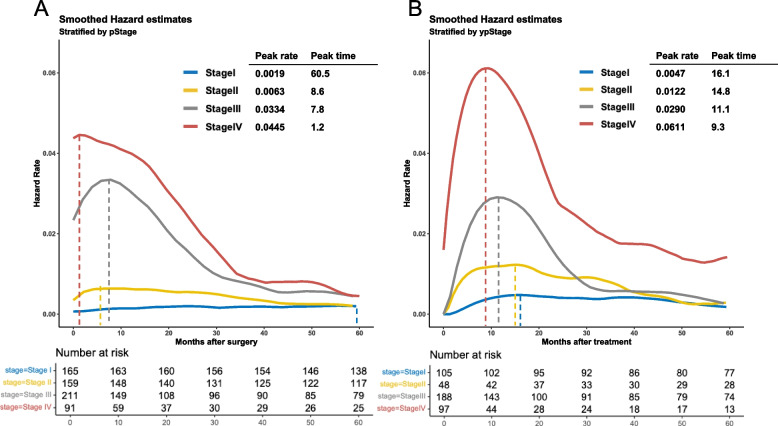
Smoothed hazard functions for recurrence stratified by TNM stage among patients with (**A**) surgery alone and (**B**) neoadjuvant therapy followed by surgery for oesophageal squamous cell carcinoma

We divided patients into two groups, those with surgery alone and those with neoadjuvant therapy followed by surgery. The latter cohort is the group of cStage II or higher OSCC with indications and tolerability for neoadjuvant therapy. Figure 5A shows the HRs of recurrence for patients who underwent surgery alone according to TNM stages. Stage III and IV patients showed a significantly early peak recurrence time, and there were no prominent HR peaks for Stage I and II. Figure 5B shows HRs for recurrence after surgery among patients with neoadjuvant surgery followed by surgery according to TNM stage. As the stage progressed, the peak HR became higher, and the peak time got shorter. Patients with ypStage IV OSCC had the highest peak rate (0.0611) at 9.3 months and higher HRs than lower stage OSCC during the entire surveillance period. The peak rate (0.029) of ypStage III OSCC was observed at 11.1 months and HRs after 30 months maintained at low value (< 0.01). From 40 months after treatment, HRs for recurrence in ypStage I, II and III OSCC patients showed almost the same value.

### Effects of Pathological Parameters on Hazard Rates of Recurrence

Patients were divided into groups according to several pathological variables of interest and HRs were plotted against time. This analysis offered visualized information regarding the effects of pathological variables on changes in HRs. The histopathological tumour response was evaluated according to the histological criteria of the Japanese Society for Oesophageal Disease [13]. In patients undergoing neoadjuvant therapy, patients with Grade0/1 therapeutic effects had a greater risks of recurrence than those with Grade2/3 (Supplemental Fig. 1A). Peak HR for patients with Grade0/1 was observed at 9.1 months after treatment, with a value of 0.0326. In contrast, peak HR for patients with Grade2/3 was slightly later at 13.2 months after surgery, with a smaller value of around 0.013. Further plots were created using histological grade in all cohorts (Supplemental Fig. 1B). The HRs for both groups increased to a peak at almost the same time from treatment (G1, GX: 8.6 months; G2–3: 8.9 months), but the HRs for patients with histological grade G2–3 were roughly twice as high as those for patients with G1 or GX tumours. In addition, we analyzed HRs for recurrence stratified by pathological lymph node metastasis status (Supplemental Fig. 1C). As for pN0, the RFS hazard curve was almost flat and consistently low HRs (less than 0.004) were observed. HRs for pN1, pN2 and pN3 peaked at almost the same time (around 11 months after treatment), although peak HRs got higher as the pN status increased. Analyzing HRs for recurrence stratified by lymph node metastasis status reveals that pathological lymph node status well reflects the prognosis for OSCC. Moreover, we analyzed hazard curves for recurrence according to the resection margin. As shown in Supplemental Fig. 1D, the peak hazard rate of the R1 group was more than three times as high as that of the R0 group (peak hazard rates: 0.0774 and 0.0194, respectively). The peak recurrence time of the R1 group was 2 months earlier than that of the R0 group (peak months: 7.29 and 9.23, respectively). This result implicates that resection margin is the solid risk factor for early recurrence.

## Discussion

Prompt detection of treatable recurrence might lead to offering salvage therapy before the tumour becomes untreatable [14]. An appropriate ﻿risk analysis of tumour recurrence is necessary to set an optimal surveillance program for each patient, whereas unnecessary examinations should be avoided in terms of costs and distress to patients and their caregivers [2]. However, because of the lack of evidence about optimal surveillance strategies, the follow-up protocols after oesophagectomy vary considerably even within a single country and reflect institutional preferences [15]. Moreover, there is no evidence-based consensus on the optimal follow-up regimen considering the recurrence risk of each stage after oesophagectomy for OC. The aim of this study was to propose a rational follow-up surveillance program based on the evaluation of the chronological changes of recurrence hazards after oesophagectomy. To the best of our knowledge, our analysis using the hazard function is the first to provide evidence regarding the optimal surveillance intensity for curatively resected OSCC.

First, in this study, we demonstrated that the HR of recurrence for an entire cohort increased steeply until less than 1 year after initial treatment, with a gradual decrease thereafter. Almost 2 years after surgery, the HR had fallen to half its maximum level. These findings are in consistent with previous studies in which the majority of recurrences occurred within 2 years after surgery [2], and rationally supports intensive surveillance within the first few years after resection, which is widely practiced but based on ambiguous evidence. Secondly, we stratified patients according to TNM stage and analyzed the chronological changes in HRs at each stage. In the analysis of HRs for OS, the peak times of OS hazard rates for stage III and IV are almost the same and the peak values of OS hazard rates showed distinct differences among each stage. Similar trends were observed in the analysis of HRs for recurrence. The results revealed that patients with higher TNM stages had a higher peak value of HR for recurrence and survival, but there were no apparent differences in the peak times. As for stage I OSCC, HRs for recurrence maintained at relatively low level through the observational period and the necessity of intensive surveillance especially in the early post-operative period is questionable. After 45 months, HRs for recurrence in stage I and II reached negligible low value. Post-operative surveillance more than 5 years for stage I and II OSCC might be unnecessary.

Furthermore, in light of worldwide current standard treatment strategy, which is neoadjuvant therapy and surgery for advanced OC, we analyzed the cohort of patients with neoadjuvant therapy followed by surgery. This cohort consists of cStage II or higher OSCC with tolerability of neoadjuvant chemotherapy and lacks heterogeneity of patient background. We stratified this cohort according to ypStage defined by the eighth edition of TNM classification [10]. The analysis of the cohort of patients with neoadjuvant therapy reveals that as the TNM stage increased, the HRs for recurrence showed higher peak values and shorter peak times. The results dovetail with the hypothesis that OC of higher stage recurrents more frequently and earlier. With the increasing use of neoadjuvant therapy for OC, the current TNM staging system separates classifications into pathological (pStage) and post-neoadjuvant pathological (ypStage) groups [16] {Rice, 2016 #43}. Our findings indicated that the classification of ypStage accurately reflects the prognosis for OSCC patients who undergo neoadjuvant therapy and surgery in terms of recurrence hazard.

The current NCCN guidelines recommend intensive surveillance for Stage II and III OC patients after trimodal therapy within the first 3 years after resection [4]. In our study, the HR for recurrence of ypStage II and III remained at a relatively high level until 40 months after treatment. These data roughly support the surveillance strategy of the NCCN guidelines for ypStage II and III OSCC patients. Meanwhile, the HR for recurrence of ypStage I showed no prominent peak and was maintained at a relatively low level. Comparing pStage I patients who underwent surgery alone and ypStage I patients who received neoadjuvant therapy and surgery, ypStage I patients showed slightly higher HRs than pStage I for five years after treatment. This difference is probably due to downstaging by neoadjuvant therapy, however both curves showed no distinct peak and maintained at low value. Consequently, Stage I OSCC including ypStage I might not require intensive surveillance from first year after treatment.

In this study, we investigated post-operative surveillance period and intensity after oesophagectomy. However, optimal surveillance method remains unclear. Lou et al. highlighted that CT scans are effective at identifying subclinical recurrences, but upper endoscopy rarely detects subclinical recurrences in survivors of OC [2]. Recently, in patients with curatively resected primary OC, second primary cancers such as gastric conduit cancers and tumours in the upper aero digestive tract have gained recognition. Not only in Asian countries including Japan, but also in Western areas, the incidence of second primary cancers after treatment of OC is high [17, 18]. Considering the need for the detection of second primary cancers in patients with OC, periodic upper endoscopy for patients even with low risk of recurrence might be necessary for the entire lifetime to detect second primary cancers at early stage. We recommend annual gastroscopy as a follow-up for all postoperative patients.

We have argued for the necessity of more intensive surveillance for the patients with higher stage OSCC. However, considering the cost, decreasing the quality of life, and potential hazards of radiation exposure that accompany of many of the surveillance studies, comparing the efficacy of different follow-up protocols with an analysis of such factors would be an important next step in the management of patients with OSCC.

This study has several limitations. First, it is retrospective study from a single high-volume center experience and the data may not be generalizable. Moreover, despite our relatively aggressive surveillance protocol, it is possible that some recurrences might be missed and not identified timely. A protocol including a strictly aggressive surveillance strategy can answer this question. An ideal study would be a prospective randomized study comparing an aggressive surveillance approach versus a lessened approach (for example, studies performed only when a patient complains of a symptom). However, we do not hear the launch of that kind of study. Second, ﻿because we only analyzed OSCC, which is the predominant histological type of OC in Asian countries including Japan, the results might not be extrapolated to oesophageal adenocarcinoma, which is the major type of OC in Western countries. The oncological behaviour of oesophageal adenocarcinoma is totally different from OSCC. Third, during this study period, there were drastic changes in the therapeutic strategy for advanced OC. Since 2007, neoadjuvant therapy followed by surgery is the standard treatment for advanced OSCC in Japan. The cohort of this study included patients who underwent upfront surgery and patients who received neoadjuvant therapy and subsequent surgery. To eliminate the effect of heterogeneity, we analyzed the cohorts of patients separately. The sample sizes of each cohort were relatively small, and the statistical power of analysis seemed to be somewhat weak. However, more distinct differences in peak rate and peak recurrence time among the patients with neoadjuvant therapy were observed. Our analyses using the hazard function could provide the first evidence of chronological changes in the recurrence risk of curatively resected OSCC and confirmed that the dynamics of the HR differed significantly by OSCC stage. Our results may contribute to establishing appropriate surveillance programs in future clinical trials.

## Conclusion

In summary, the transition of HRs in the recurrence of curatively resected OSCC showed distinct patterns by stage stratified by TNM classification. Our results support intensive surveillance within the first 3 years after surgery in Stage III OSCC and within the first 4 years in Stage IV OSCC, followed by subsequent annual screening. Meanwhile, a reduction in the surveillance intensity might be justified in Stage I OSCC patients, regardless of whether neoadjuvant therapy is administered.

## Supplementary Information

**Additional file 1.**

## Data Availability

The data that support the findings of this study are available from the corresponding author, HD, on reasonable request.

## Abbreviations

OC: Oesophageal cancer
OSCC: Oesophageal squamous cell cancer
NCCN: National Comprehensive Cancer Network
OS: Overall survival
RFS: Recurrence-free survival
TNM: Tumour-node-metastasis
