# Supplementary material for: Surveillance Strategy after Curative Resection for Oesophageal Squamous Cell Cancer Using the Hazard Function
Source: BMC Cancer. 2022 Dec 1;22:1245. doi: 10.1186/s12885-022-10345-5 (PMC9716854; doi:10.1186/s12885-022-10345-5)
Supplement: Supplementary file 1 — Additional file 1. [file 12885_2022_10345_MOESM1_ESM.pptx]

## Slide 1
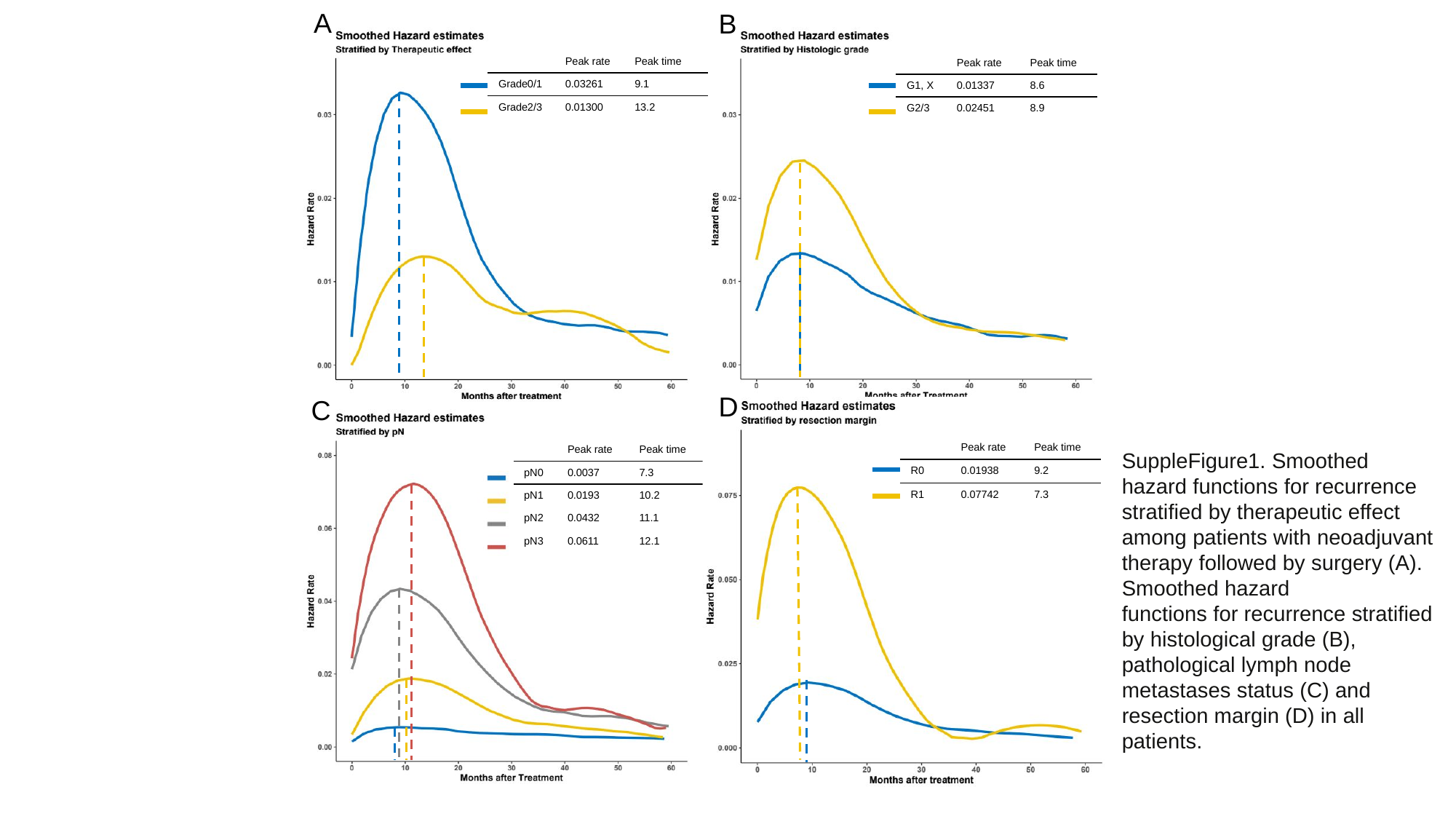

A
B
| | Peak rate | Peak time |
| --- | --- | --- |
| Grade0/1 | 0.03261 | 9.1 |
| Grade2/3 | 0.01300 | 13.2 |
| | Peak rate | Peak time |
| --- | --- | --- |
| G1, X | 0.01337 | 8.6 |
| G2/3 | 0.02451 | 8.9 |
D
C
| | Peak rate | Peak time |
| --- | --- | --- |
| R0 | 0.01938 | 9.2 |
| R1 | 0.07742 | 7.3 |
| | Peak rate | Peak time |
| --- | --- | --- |
| pN0 | 0.0037 | 7.3 |
| pN1 | 0.0193 | 10.2 |
| pN2 | 0.0432 | 11.1 |
| pN3 | 0.0611 | 12.1 |
SuppleFigure1. Smoothed hazard functions for recurrence stratified by therapeutic effect
among patients with neoadjuvant therapy followed by surgery (A). Smoothed hazard
functions for recurrence stratified by histological grade (B), pathological lymph node metastases status (C) and resection margin (D) in all patients.
